# Steroid-Refractory Chronic Idiopathic Thrombocytopenic Purpura Responding to Combination Therapy With Eltrombopag and Rituximab

**DOI:** 10.7759/cureus.10305

**Published:** 2020-09-08

**Authors:** Mohanad Ahmed, Mohamed A Yassin, Elabbass Abdelmahmuod

**Affiliations:** 1 Internal Medicine, Hamad Medical Corporation, Doha, QAT; 2 Hematology and Oncology, Hamad General Hospital, Doha, QAT

**Keywords:** itp, eltrombopag, rituximab, steroid-refractory, combination therapy

## Abstract

Idiopathic thrombocytopenic purpura (ITP) is a disease in which the immune system attacks platelets and decreases their number, which increases the patient's risk of bleeding. ITP is diagnosed by exclusion and usually manifests as acute disease. It is self-limiting in pediatric patients, while it tends to be a chronic disease in adults. Treatment of ITP focuses on maintaining a sufficient platelet count to decrease the risk of bleeding rather than normalize the platelet count. Most patients respond to first-line treatments, such as steroids and intravenous immunoglobulin (IVIG). However, some cases can become steroid-dependent or unresponsive to first-line therapy, in which case, second-line therapy is required to control symptoms or the platelet count. Second-line therapy includes either rituximab or a thrombopoietin receptor agonist (eltrombopag, romiplostim). In a few cases, when second-line therapy alone is insufficient to control the disease, a combination of therapies is required to control the symptoms and platelet count. Here, we present a case of a 41-year-old man with refractory ITP who did not respond to first-line treatment with either steroids or IVIG or a combination of the two, and also did not respond to eltrombopag alone and required a combination of eltrombopag and rituximab to control his disease.

## Introduction

Immune thrombocytopenia (ITP, also called idiopathic thrombocytopenic purpura or immune thrombocytopenic purpura) is an acquired thrombocytopenia caused by autoantibodies against platelet antigens [1]. Thrombocytopenia may be inherited or acquired. The causes of thrombocytopenia can be classified into three groups: diminished production (caused by viral infections, vitamin deficiencies, aplastic anemia, or drugs), increased destruction (caused by drugs, heparin [heparin-induced thrombocytopenia], idiopathic disease, pregnancy, immune system), and sequestration (caused by an enlarged spleen, pregnancy, or childbirth) [2]. With a low platelet count, the patient will be at risk for severe and life-threatening bleeding, although the overall risk of bleeding in ITP is low. Bleeding risk is highest in individuals with a platelet count of less than 10,000/mm^3^ [3]. ITP can be primary due to autoimmune mechanisms that lead to platelet destruction, platelet underproduction that is not triggered by an apparent associated condition, or secondary to other conditions, diseases, or medications. Persistent ITP is defined by a duration of 3-12 months since the diagnosis, while the chronic state is defined by 12 months or more that have elapsed since the diagnosis [4]. Because ITP is often a chronic disease in adults, the prevalence exceeds the incidence. Chronic refractory ITP may be defined as the failure of any modality to maintain the platelet count above 20,000/mm^3^ for an appreciable time without unacceptable toxicity [5,6].

ITP is diagnosed by exclusion, and there are different modalities of treatment. The goal of ITP therapy is to reduce the risk of clinically important bleeding. Thus, many patients do not require interventions to increase the platelet count. The need for intervention is guided by bleeding symptoms and the platelet count (i.e., whether it is sufficiently low to confer severe bleeding risk). First-line therapy includes steroids and intravenous immunoglobulin (IVIG). Some patients may not respond well to glucocorticoids or IVIG after treatment, so second-line therapy with splenectomy, rituximab, thrombopoietin receptor agonists (TPO-RAs), or immunosuppressive therapy is used to achieve the desired response.

Before initiating ITP therapy, we performed screening tests for HIV and hepatitis C virus infection. Hepatitis B testing is necessary for patients who will receive rituximab and may be performed earlier. Screening for Helicobacter pylori may also be appropriate. Patients should be current with all recommended routine immunizations, and immunizations for encapsulated organisms should be provided for patients who are likely to undergo splenectomy to avoid exacerbations or relapse of these infections.

Herein, we report our experience in treating a young adult who was diagnosed with chronic refractory ITP. He did not respond to different first-line therapies, nor did he respond to numerous combined second-line therapies. However, there was a rapid increase in his platelet count and improvement in his symptoms after the initiation of combination therapy consisting of eltrombopag (EPAG) and rituximab, which did not cause any significant side effects.

## Case presentation

We present a 41-year-old man who had a history of Hodgkin lymphoma (HL). He developed HL when he was 16 years old, was treated with radiation therapy for five months, and he subsequently remained disease-free since that time. He presented to us on February 12, 2017 with ecchymosis and skin rash over most of his body. Clinical examination was only significant for ecchymosis and rash over the trunk and upper limbs, with a non-itchy rash scattered throughout his body. General laboratory tests were requested for the patient, which revealed a platelet count of 3,000/mm^3^. Other laboratory tests, including hemoglobin, respiratory function test, liver function test, coagulation profile, inflammatory markers, viral serology, and autoimmune workup, were all within reference limits. Peripheral smear was performed, and it showed severe thrombocytopenia. Thus, a diagnosis of ITP was made by exclusion, and the patient was admitted to the hospital for first-line therapy. The patient received IVIG and steroid for four days, and his platelet count increased to 112,000/mm^3^. He was subsequently discharged as an outpatient and instructed to take oral prednisolone 80 mg daily, with follow-up to taper the use of the steroid.

One week later, the patient again presented for the second time with the same skin rash and ecchymosis, although he continued to take his medication. Laboratory tests showed a platelet count of 5,000/mm^3^, and the patient was admitted once more and was diagnosed with ITP by exclusion. Administration of IVIG resumed, and pulsed steroid was administered for three days, after which his symptoms improved, and his platelet count increased to 32,000/mm^3^. Then, he was discharged again, with the continuation of oral steroid.

**Figure 1:**
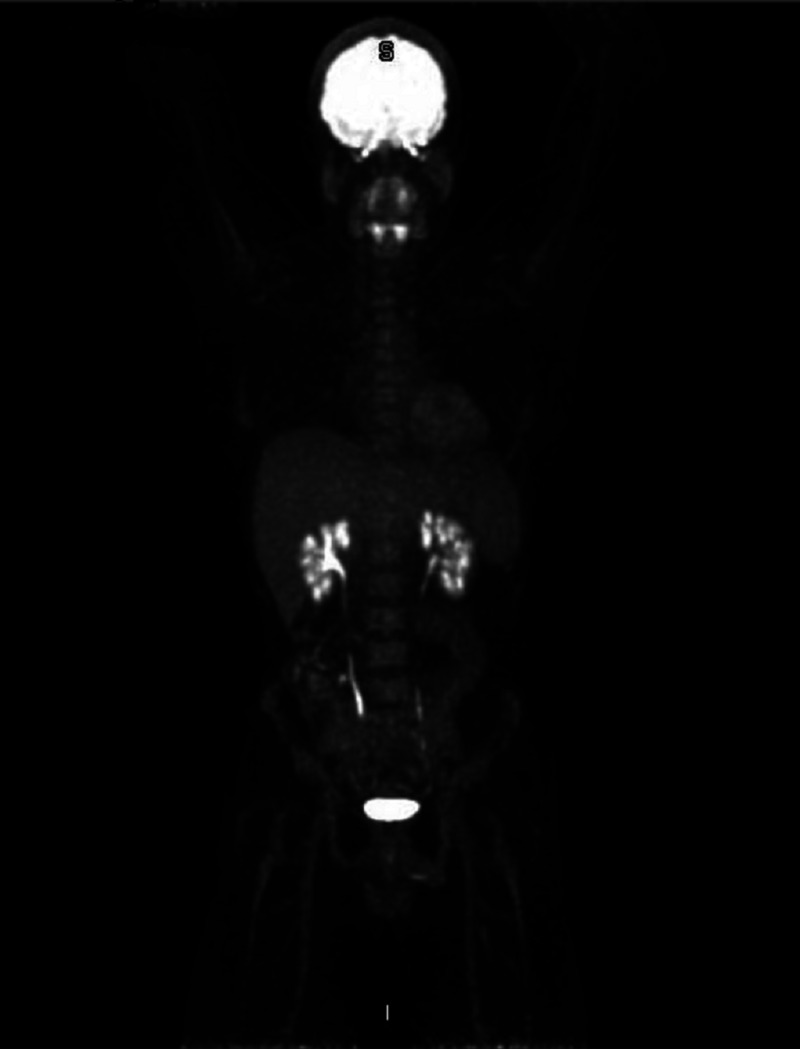
Positron emission tomography (PET) scan shows no sign of lymphoma relapse, or any other fluorine 18 fluorodeoxyglucose (FDG)-avid malignancy was found.

Five days after the second discharge, he presented again with ecchymosis and platelet count of 3,000/mm^3^, was admitted to the hospital as an ITP case, and IVIG and steroid were resumed. This time, a positron emission tomography (PET) scan was performed to rule out a relapse of lymphoma (Figure 1). The PET scan was negative for malignancy. During this third admission, the decision was made to administer second-line treatment. Therefore, EPAG 75 mg daily was initiated on March 8, 2017, and a bone marrow study was performed to detect the presence of any concomitant hematological disease. One week after hospital admission, the patient’s symptoms improved, but his platelet count remained at 4,000/mm^3^. On March 17, 2017, the patient was discharged on EPAG 75 mg, with close follow-up and observation due to low platelet count.

On outpatient follow-up on March 21, 2017, the bone marrow analysis indicated the presence of hypercellular bone marrow in addition to erythroid and megakaryocytic hyperplasia. His platelet count remained at 4,000/mm^3^. Therefore, we decided to admit the patient for the fourth time and administer combination therapy with another cycle of IVIG, pulsed steroid, and EPAG.

On March 24, 2017, the patient was admitted to the hospital for the fourth time with a platelet count of 5,000/mm^3^, and three medications consisting of IVIG, pulsed steroid, and EPAG were administered. After five days of this combination, the platelet count remained at 5,000/mm^3^, and the patient was discharged because he was asymptomatic. Close follow-up in the outpatient clinic was planned after a course with the combination of EPAG and oral steroid.

The patient was examined in the outpatient clinic on April 4, 2017, and his platelet count was 5,000/mm^3^. At that time, we considered switching the patient to the combination therapy of rituximab and EPAG. After being informed of the benefits and risks of this combination, the patient agreed to undergo this therapy. We subsequently scheduled four cycles of weekly intravenous rituximab, and the patient continued with oral EPAG. On April 5, 2017, he received the first cycle of rituximab, which was well tolerated. One week later, he received the second cycle, and his platelet count increased to 47,000/mm^3^. After completing four cycles, his platelet count increased to 200,000/mm^3^. Then, he received only EPAG 75 mg daily.

**Figure 2:**
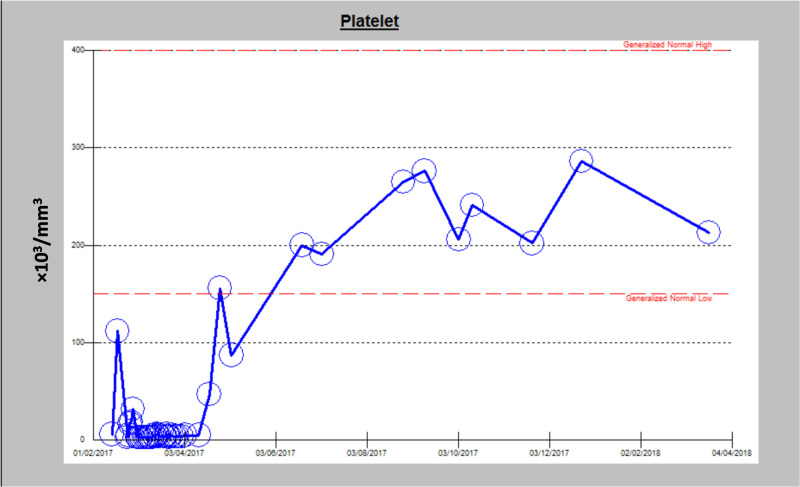
Change in platelet count is shown before, during, and after treatment.

One month later, the patient was examined in the outpatient clinic, and a platelet count of 191,000/mm^3^ was measured. Therefore, the EPAG dose was reduced to 50 mg daily. Over the following months, the patient follow-up indicated a maintained platelet count over 200,000/mm^3^. Figure 2 shows the platelet count before, during, and after treatment.

## Discussion

In most cases, ITP responds to first-line treatment with corticosteroids or IVIG. The advantages of corticosteroids are their effectiveness, availability, and lower costs compared to IVIG. Corticosteroids are usually used in non-life-threatening ITP. However, IVIG is very effective in patients with critical bleeding or in those who do not respond to corticosteroid treatment [7].

There are three principal choices of second-line treatment: splenectomy, rituximab, and TPO-RAs, and they differ in their mechanism of action, efficacy, and risks [8]. In selecting treatment regimens for the management of ITP, it is important to customize care based on duration, type, adverse effects, cost, and duration of hospitalization.

Indications for second-line therapy include patients with thrombocytopenia that is associated with significant bleeding symptoms (e.g., mucosal purpura or more serious bleeding) or for patients with severe, persistent, or recurrent thrombocytopenia (e.g., platelet count <20,000/mm^3^) following glucocorticoid-based treatments. For second-line therapies, rituximab or a TPO-RA is recommended rather than chronic glucocorticoids [9].

Rituximab is an anti-CD20-directed cytolytic monoclonal antibody that inhibits B cells from producing autoantibodies as well as reverting T-cell abnormalities in patients who respond to treatment. It is used in many clinical conditions, including blood malignancies, autoimmune diseases, and post-organ transplant [10].

EPAG, an oral TPO-RA, approved for the treatment of thrombocytopenia in adults with chronic immune thrombocytopenia (cITP) and insufﬁcient response to first-line therapy, such as corticosteroids and immunoglobulins [11]. It was also reported that EPAG can induce treatment-free remission in cases of cITP [12].

Upon review of the literature regarding the relationship between HL and ITP, we found that ITP is rarely found in patients with HL [13]. Among the 4,090 HL patients of the British National Lymphoma Investigation Registry, only eight cases of ITP were found. However, to the extent of our knowledge, there have been no reported cases of ITP that develop after treatment for HL.

In our patient, the initial picture was concomitant with steroid-responsive ITP because he responded well to steroids upon the first admission. However, on subsequent admissions, he developed resistance to steroids and IVIG. Additionally, no major bleeding developed, although his platelet count was less than 5,000/mm^3^. The patient clinically was doing well apart from ecchymosis, and repeated scans showed no evidence of relapse of HL, which indicated that it is unlikely that his ITP was related to his previous cancer.

Our decision to begin second-line treatment was justified, according to the UpToDate guidelines for the management of ITP, as the patient’s condition was that of severe, persistent, or recurrent thrombocytopenia (e.g., platelet count <20,000/mm^3^). Although many patients respond to second-line monotherapy with EPAG or combined therapy consisting of steroid and EPAG, our patient continued to exhibit a low platelet count after undergoing combined therapy.

A literature search for recommendations for the treatment of refractory cases revealed few reports that encourage the use of EPAG-based combinations. Therefore, we decided to initiate rituximab cycles in addition to EPAG for our patient after we discussed the benefits and risks with the patient.

After receiving two cycles of rituximab, the patient’s platelet count significantly increased and returned to normal after completing four cycles. Then, the patient maintained normal platelet counts even after we reduced the dose of EPAG from 75 to 50 mg daily. Follow-up of the patient indicated that his platelet counts did not decrease again, which previously occurred for every course of treatment prior to the administration of the combination of rituximab and EPAG.

## Conclusions

Although most cases of ITP respond well to first-line treatment, some rare cases can pose a dilemma by not responding or being refractory to first-line treatment. Herein, we share our experience in treating a case of steroid-refractory chronic ITP that responded well to combination therapy with EPAG and rituximab when first-line therapy and single second-line therapy fail to improve the patient. This article will assist other practitioners in selecting the appropriate second-line therapy combinations in such situations.
